# Microcystin Toxicokinetics, Molecular Toxicology, and Pathophysiology in Preclinical Rodent Models and Humans

**DOI:** 10.3390/toxins13080537

**Published:** 2021-07-29

**Authors:** Tarana Arman, John D. Clarke

**Affiliations:** Department of Pharmaceutical Sciences, Washington State University, Spokane, WA 99202, USA; tarana.arman@wsu.edu

**Keywords:** microcystin, toxicokinetics, pathophysiology, multifactorial exposure, nonalcoholic fatty liver disease (NAFLD)

## Abstract

Microcystins are ubiquitous toxins produced by photoautotrophic cyanobacteria. Human exposures to microcystins occur through the consumption of contaminated drinking water, fish and shellfish, vegetables, and algal dietary supplements and through recreational activities. Microcystin-leucine-arginine (MCLR) is the prototypical microcystin because it is reported to be the most common and toxic variant and is the only microcystin with an established tolerable daily intake of 0.04 µg/kg. Microcystin toxicokinetics is characterized by low intestinal absorption, rapid and specific distribution to the liver, moderate metabolism to glutathione and cysteinyl conjugates, and low urinary and fecal excretion. Molecular toxicology involves covalent binding to and inhibition of protein phosphatases, oxidative stress, cell death (autophagy, apoptosis, necrosis), and cytoskeleton disruption. These molecular and cellular effects are interconnected and are commonly observed together. The main target organs for microcystin toxicity are the intestine, liver, and kidney. Preclinical data indicate microcystins may also have nervous, pulmonary, cardiac, and reproductive system toxicities. Recent evidence suggests that exposure to other hepatotoxic insults could potentiate microcystin toxicity and increase the risk for chronic diseases. This review summarizes the current knowledge for microcystin toxicokinetics, molecular toxicology, and pathophysiology in preclinical rodent models and humans. More research is needed to better understand human toxicokinetics and how multifactorial exposures contribute to disease pathogenesis and progression.

## 1. Introduction

Photoautotrophic cyanobacteria contributed to the creation of Earth’s aerobic environment approximately 2.4 billion years ago in the Great Oxidation Event [1,2,3,4]. In Earth’s current environment, cyanobacteria, also known as blue-green algae, are ubiquitous in freshwater and marine environments and form dense blooms under favorable conditions [3,5,6]. Over the last 70 years, the frequency of cyanobacterial blooms has increased in approximately 60% of the lakes in North America and Europe due to the eutrophication of aquatic ecosystems and increased global temperatures [3,6,7,8]. 

Cyanobacteria have a range of positive or negative ecological and biological effects [9,10,11,12]. As a bioresource, cyanobacteria have a high biomass yield and are used as an effective bio-fertilizer [13]. Furthermore, primary cyanobacterial metabolites such as phenolics, free fatty acids, and phytohormones are being investigated for their antibiotic, immunosuppressant, anti-cancer, and anti-inflammatory activities [11,12]. However, cyanobacteria can cause an unpleasant taste and/or odor in both drinking and recreational water because they produce compounds such as geosmin and 2-methylisoborneol [14]. Cyanobacterial overgrowth also compromises water clarity and oxygen availability [6,7], which may result in large-scale fish and other macrophyte mortality caused by detrimental effects to their aquatic habitats. Many cyanobacterial species also produce harmful secondary metabolites, commonly known as cyanotoxins [3,8,12,15]. Cyanotoxins are broadly grouped as hepatotoxins, neurotoxins, and dermatoxins [12,16,17]. Table 1 summarizes common cyanotoxins, the cyanobacteria that produce them, their mode of action, and their toxic effects. This review focuses on microcystins, a group of cytotoxic and carcinogenic hepatotoxins [15,18,19,20].

**Figure 1:**
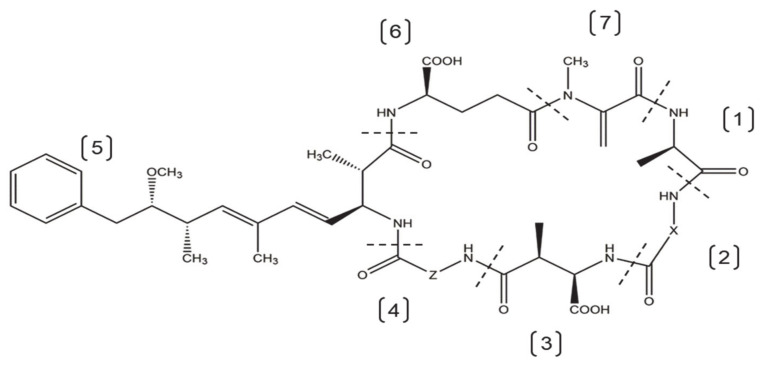
The general cyclic structure of microcystins. The heptapeptide contains the following amino acids: [1] D-Alanine (D-Ala); [2] Variable L-amino acid (X); [3] D-Methyl aspartic acid (D-MeAsp); [4] Variable L-amino acid (Z); [5] 3-amino-9-methoxy-2,6,8-trimethyl-10-phenyldeca-4,6-dienoic acid (Adda); [6] D-Glutamic acid (D-Glu); [7] N-Methyldehydroalanine (Mdha).

An extensive review of worldwide cyanobacterial blooms found that microcystins are the most commonly reported cyanotoxins in freshwater [8]. Microcystins are monocyclic heptapeptides (Figure 1) [22,23]. There are more than 240 microcystin variants that are defined by the different X and Z amino acids at positions 2 and 4 of the heptapeptide and methylation status of D-Methyl aspartic acid (D-MeAsp) and *N*-Methyldehydroalanine (Mdha) (Figure 1) [24,25]. The variable amino acids in the cyclic chain primarily determine the polarity of the microcystin variant, and the 3-amino-9-methoxy-2,6,8-trimethyl-10-phenyldeca-4,6-dienoic acid (Adda) side chain has been implicated in the inhibition of serine-threonine protein phosphatase activity [24,26,27,28]. Microcystin-leucine-arginine (MCLR) is the most common and possibly one of the most toxic variants found in the environment [18,22]. Although there are less common microcystin variants with similar toxicological effects (e.g., MCRR, MCYR, and MCLA) [22,29], MCLR has the most abundant toxicological data and is the only variant with human exposure guidance information. The World Health Organization (WHO) has issued a provisional guidance value of 1 µg/L for lifetime drinking water, which equates to a Tolerable Daily Intake (TDI) level of 0.04 µg/kg bodyweight [30,31]. It is important to note that the toxicokinetics, molecular toxicology, and pathophysiology data for the many different microcystin variants are incomplete. However, WHO has recommended methods to evaluate guidance values for other microcystins based on the existing guidance values for MCLR [31]. Throughout this review, specific microcystin variants are discussed and generalized statements are made, but more research is needed in many areas to understand microcystin effects more fully on human health. 

## 2. Human Microcystin Exposure

Sources for human microcystin exposures include consuming drinking water, fish, shellfish, vegetables, and algal dietary supplements, as well as being exposed through recreational activities [32,33,34,35]. Based on the WHO TDI guidelines of 0.04 µg/kg for MCLR, the safe estimated daily intake (EDI) for a 60 kg adult is 2.4 µg/day [36]. Multiple studies indicate microcystin EDI values may exceed the TDI after consumption of contaminated water, fish, and vegetables. For example, in populations who consumed contaminated fish and water from lakes in North America and Africa, all but one population were estimated to exceed the TDI, with one population having an EDI of 180 µg/day [37]. Another study calculated an EDI of 4.7 µg/day for people who consumed 300 g of fish from Southeast Asian aquaculture farms [36]. In addition, crop irrigation from contaminated water sources was reported to introduce microcystins into vegetables [38]. Another study from southern China reported high concentrations of MCLR, MCRR, and MCYR in vegetables, with almost 60% of the vegetables posing a moderate to high health risk after consumption [35]. Another study quantified MCLR in four varieties of leafy vegetables after application of cyanobacterial manure and calculated an EDI ranging from 0.18–1.32 µg/day [39]. These data illustrate the risk of human microcystins exposure at multiple trophic levels of the food web caused, in part, by bioaccumulation in fish, shellfish, and vegetables [8,34,35,40,41,42]. Exposure to microcystins through drinking water and reports of liver and gastrointestinal stress has been documented by several groups [43,44]. Chronic microcystin exposure through drinking water has also been linked to various adverse health effects such as increased incidences of liver cancer and other chronic liver ailments [8].

An incidence of microcystin toxicity after consumption of algal dietary supplements was reported in 2018 in a 67-year-old lung cancer patient [45]. The patient was self-medicating with natural products and presented at the emergency department with acute toxic hepatitis. The patient was taking multiple prescription medications and dietary supplements, including *Chlorella*. Chemical characterization of the *Chlorella* supplement revealed 1.08 µg of MCLR per gram of *Chlorella* biomass, which may have contributed to the acute hepatitis in this patient. 

Acute toxicity after accidental exposure to high concentrations of microcystins during recreational activities has been reported. Accidental ingestion, dermal contact, and inhalation of aerosolized cyanotoxins are common routes of microcystin exposure during recreational activities such as swimming, water-skiing, etc. [32,46,47,48]. In January 2015, environmental monitoring of water quality at a beach in Montevideo, Uruguay detected microcystin levels ranging between 2.9–8.2 mg/L, well above the WHO guidelines for microcystin in recreational water which ranges from 2–20 µg/L [49,50]. A 20-month-old girl suffered gastrointestinal symptoms (diarrhea and vomiting) within a few hours of recreation at the beach and eventually developed fatigue and jaundice. The girl progressed towards a liver failure condition and required a liver transplant [49]. Analysis of the resected liver revealed the presence of MCLR (2.4 ng/g of tissue) and [D-Leu^1^] MCLR (75.4 ng/g of tissue) [49]. Another acute case of cyanobacterial poisoning was reported from Salto Grande Dam, Argentina in January 2007. Algal blooms were present, and MCLR concentrations were measured to be 48.6 µg/L. A 19-year-old man who was jet-skiing and frequently in and out of the water for approximately 2 h, developed gastrointestinal symptoms (e.g., vomiting) and muscle weakness a few hours post-exposure. Three days later the patient was admitted for intensive care due to respiratory distress and elevated serum markers of hepatic and renal damage. Fortunately, the man recovered 20 days post-exposure [51]. 

A tragic incident of human fatalities after exposure to microcystins occurred in a dialysis clinic in Caruaru, Brazil in 1996 when patients received dialysis fluid contaminated with microcystins, leading to the death of 60 patients [52]. Analysis of both the serum and the liver samples confirmed the presence of three microcystin variants in these patients: MCLR, MCYR, and MCAR. The concentration of microcystins in serum ranged from 1 to 10 ng/mL and in liver samples ranged from 0.1 ng/mg to 0.5 ng/mg liver tissue [52]. These examples illustrate the major microcystin exposure routes in humans and emphasize the need to understand microcystin toxicokinetics and toxicology. 

## 3. Microcystin Toxicokinetics

**Figure 2:**
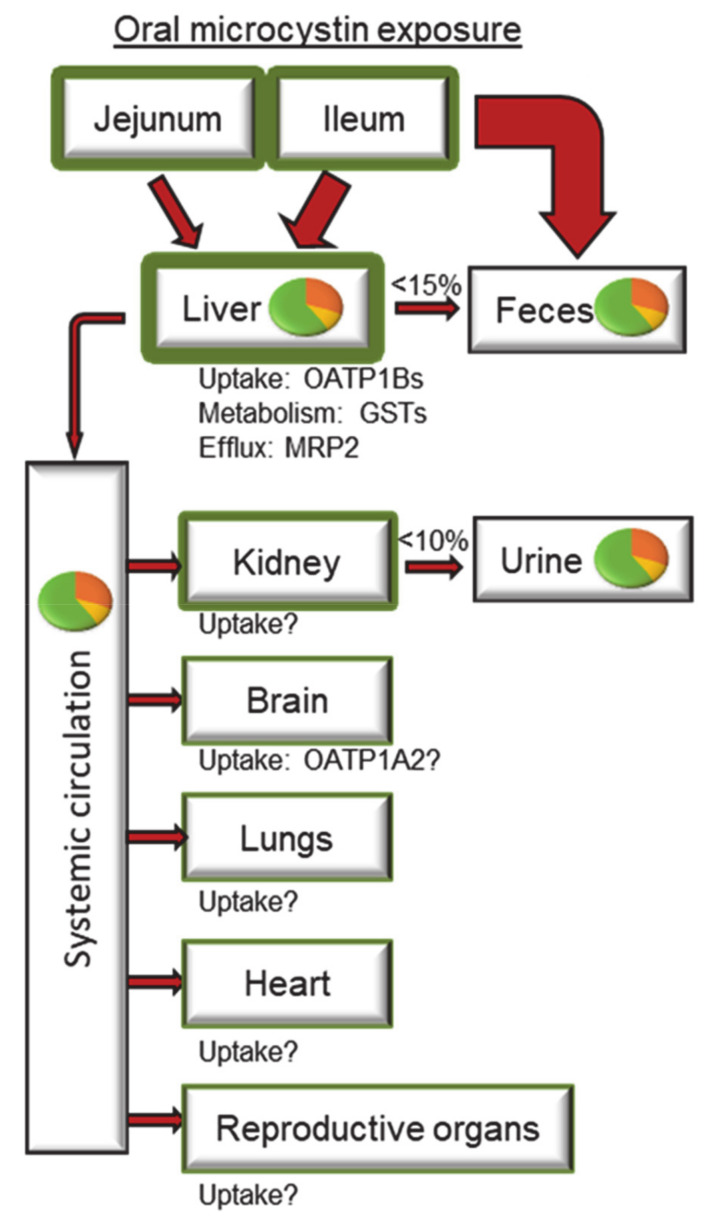
Microcystin absorption, distribution, metabolization, and excretion after oral exposure. Data from preclinical models indicate most of the microcystin remain in the intestinal contents and is eliminated into the feces. More microcystin is absorbed through the ileum than the jejunum. The intestine and the liver are the major target site of distribution (thick green borders), followed by the kidney, then other organs. Within the liver, systemic circulation, feces, and urine, the parent microcystin (green in the pie chart) is the most abundant form, followed by microcystin-cysteine (orange), then microcystin-glutathione (yellow). Less than 15% and 10% of the total microcystin is excreted into the feces and urine, respectively. Abbreviations: OATP1Bs: organic anion transporting polypeptide 1B isoforms; GSTs: glutathione-S-transferase; MRP2: multidrug resistance associated protein 2; OATP1A2: organic anion transporting polypeptide 1A2.

Multiple studies have now reported microcystin concentrations in human serum up to 1.8 ng/mL, illustrating systemic exposure in different populations [53,54,55,56,57]. Like many other environmental toxins, it is difficult to obtain clear and thorough microcystin toxicokinetic data in humans, and preclinical data play a major role in determining various aspects of microcystin absorption, distribution, metabolism, and excretion. Microcystins are large cyclic heptapeptides requiring transporters to traverse cell membranes making those transporters important mediators of microcystin toxicokinetics. Using in vitro expression systems, it was shown that the organic anion transporting polypeptides, specifically, OATP1B1, OATP1B3, OATP1A2, and Oatp1b2 are responsible for the uptake of microcystins and multidrug resistance-associated protein 2 (MRP2) is responsible for its efflux [58,59,60]. Interestingly, several cell lines that do not express these OATP variants have also exhibited microcystin uptake, suggesting that there are other microcystin uptake transporters that have not been identified [61,62]. The following section summarizes what is known regarding microcystin toxicokinetics and important gaps that remain to be addressed (Figure 2). 

### 3.1. Absorption

Oral consumption is a major route of microcystin exposure, and multiple in vitro and in vivo experiments indicate low intestinal absorption for microcystins. Using Caco-2 cells to characterize the apical-to-basolateral transport, rapid uptake of MCLR from the apical side was reported (24 to 40%), but only a fraction reached the basolateral compartment (0.3–1.3%) [63]. A similar low permeability was recently reported for MCRR [64]. These data indicate efficient uptake at the apical membrane but poor basolateral efflux, with the latter observation being the driving mechanism for low intestinal permeability. Another study compared MCLR and MCRR sub-cellular localization in Caco-2 cells and reported that the two congeners have similar uptake profiles suggesting the difference in liver toxicity between the congeners is likely related to hepatic OATP substrate selectivity rather than intestinal absorption [65,66]. Although OATP2B1 is the major OATP transporter in the intestine, previous experiments demonstrated MCLR is not a substrate for this transporter [59]. Zeller et al. suggested OATP3A1 and OATP4A1 as possible candidates for intestinal uptake of MCLR and MCRR [65]. However, more research is required to identify the intestinal uptake transporters of microcystins.

Data from preclinical in vivo models provide a clearer picture of microcystin absorption. An early study in mice reported less than 2% of radiolabeled dihydro-MCLR in tissues six hours after oral administration with the highest percentage in the liver (~0.68%) followed by the small and large intestines (~0.25% each), suggesting low oral bioavailability. Interpretation of these data is limited because only ~38% of radiolabeled dihydro-MCLR remained in the gastrointestinal tract contents, making ~60% of the dose unaccounted for in this study [67]. An in situ MCLR intestinal perfusion study in Sprague Dawley rats demonstrated that more liver damage occurred after ileal perfusion compared to jejunal perfusion, suggesting greater MCLR absorption may occur in the ileum [68]. Unfortunately, this study did not measure microcystin concentrations, therefore, only qualitative conclusions can be made. Another study exposed pigs to MCLR through gavage at the TDI (0.04 µg/kg) and 50 times the TDI (2 µg/kg) [69]. No MCLR was detected in the serum of either dose group, but half of the animals in the 2 µg/kg animals accumulated ~1.1% of the total MCLR dose in the liver. The MCLR detected in the liver was covalently bound to protein phosphatases and some unbound MCLR was detected in the large intestine and the kidneys. 

Overall, these preclinical data suggest low intestinal absorption, but the data are not robust and may not be directly translatable to human populations because of limitations in transporter function in intestinal cell lines and species differences in OATPs. A comparison of published data for MCLR serum concentrations suggests a potential difference in MCLR intestinal absorption between preclinical species and humans. A study in fishermen exposed to TDI levels of MCLR (~0.04 µg/kg) reported an average MCLR serum concentration of 0.39 ng/mL. For this study and other serum MCLR studies, the time between MCLR exposure and blood collections are unknown, and the data likely represent concentrations at different times along the plasma concentration–time curve. In comparison, a study in rats reported ~2 ng/mL serum concentrations 120 min after intravenous 20 µg/kg MCLR [70]. Considering the 500-fold lower dose in oral (fishermen) versus intravenous (rats) exposure routes, it would be expected that the fisherman would have substantially lower serum concentrations than the rats, but there was only a five-fold difference between the serum concentrations. In addition, the above-mentioned study in pigs used a dose 50 times above the TDI (2 µg/kg) but did not detect any MCLR in the serum, which contrasts with the fishermen study, suggesting these animals may have a lower intestinal absorption compared to humans. Improved cell models and/or humanized preclinical species are required to definitively determine human microcystin oral absorption and bioavailability. 

### 3.2. Distribution

Data from preclinical species clearly show the liver is the primary site for microcystin distribution [71,72,73]. Intraperitoneal administration of radiolabeled dihydro-MCLR in mice resulted in 71.5% of the total radioactivity accumulated in the liver after one hour [67]. Similar results were reported by Robinson et al. after intravenous administration of MCLR in mice where 67% of the dose accumulated in the liver after 1 h [74]. In contrast, two hours after intravenous administration in Wistar rats, the highest percentage MCLR content was in muscle (17.0%), followed by liver (6.1%), kidney (3.7%), intestine (0.5%), lung (0.3%), and gonad (0.1%) [72]. Similarly, two hours after intravenous radiolabeled microcystin in albino rats, the highest percentage of microcystin content was in the liver (19.2%), gut contents (9.4%), and kidney (5.3%) [73]. Further research is needed to determine whether species differences between mice and rats explain the distribution differences observed. 

Microcystins also rapidly accumulate in the liver. Robinson et al. reported that only 25% of the dose was in the plasma one minute after intravenous administration and decreased to eight percent after three minutes, whereas the liver accumulated 23% at one minute and 56% at three minutes. The intravenous plasma half-life reported in this study was 0.8 min (alpha) and 6.9 min (beta), whereas another study reported 2.1 min (alpha) and 42 min (beta) [73]. Another study reported a half-life of 49 min (beta) [70]. Robinson et al. also reported that the percentage of the dose in the liver did not change six days post-exposure (66%). These results were later confirmed using LC-MS/MS, where the liver MCLR concentrations remained constant from two hours up to seven days [75]. These data suggest microcystins are sequestered in the liver and are not readily eliminated. This sequestration may be due to selectivity for hepatic OATP transporters and the lack of robust efflux transporters. Fischer et al. demonstrated that rodent hepatic Oatp1b2 and human hepatic OATP1B1 and OATP1B3 transport MCLR, and another study demonstrated that Oatp1b2-null mice were resistant to MCLR hepatotoxicity [59,76]. Kaur et al. demonstrated that, among seven major efflux transporters tested, only MRP2 transported MCLR [60]. Thus, efficient uptake and poor efflux may explain the preferential accumulation of microcystins in the liver. 

Species differences in plasma protein binding have been hypothesized to contribute to differences in microcystin toxicities. The LD_50_ of MCLR is 100 times lower in mammals than in fish [77]. Microcystin plasma protein binding in mammals ranged from 16 to 28%, whereas in fish it was only 4% [77]. In addition, human albumin demonstrated the highest binding rates for MCLR and MCRR compared to bovine, porcine, and several carp species [77]. In addition to the degree of protein binding, the amount of albumin content has been postulated to contribute to species differences in microcystin toxicities because humans have greater albumin content than fish. Recent evidence from the OATP research field demonstrated “facilitated dissociation,” or increased rates of uptake, when substrates were bound to albumin, supporting this species difference hypothesis [78]. Further in vitro research is needed to determine whether protein binding increases microcystin uptake by OATPs. 

### 3.3. Metabolism

Microcystin metabolism is an important aspect of microcystin detoxification. Glutathione (GSH) conjugation by glutathione-S-transferases (GSTs) is the major metabolism pathway for microcystins and produces more polar and soluble metabolites for excretion [79]. The GSH conjugate is further metabolized to a cysteinyl (Cys) conjugate, which is the major metabolite observed in vivo [79,80,81]. The Mdha group in microcystins acts as the nucleophilic center and is crucial for GSH conjugation [79]. Human GSTT1 and GSTA1 have the highest catalytic efficiency for MCLR (0.0022 and 0.0012 µM^−1^ min^−1^) [82]. It is interesting to note that the parent form of MCRR was detected in plasma, tissues, urine, and feces after intraperitoneal injection of the GSH form of MCRR, indicating in vivo conversion back to the parent form [83]

Parent microcystins are the major form found in the liver. LC-MS/MS quantification is the most reliable method for the analysis of microcystin metabolites. One study performed in rats reported MCLR as the major form in the liver for the first 24 h after administration, but it decreased to approximately 50% of the total MCLR in the liver from days three to seven [75]. Another group used LC-MS/MS and reported that MCLR was still the major form in the liver two days after oral administration in rats [84]. In contrast, an early study by Robinson et al. reported that 83% of the radiolabel in the liver was covalently associated with cytosolic components at 24 h, although this likely contains MCLR metabolites as well as MCLR conjugated to protein phosphatases [74]. Inhibition of GSH synthesis in rats created higher MCLR liver concentrations, but, interestingly, had no effect on the GSH and Cys conjugate concentrations [85].

There is clear evidence that the GSH and Cys conjugates are less toxic than the parent microcystins. For example, the GSH and Cys conjugates of MCLR were 3 to 10 times less potent inhibitors of protein phosphatase than the parent form [86]. In addition, Kondo et al. reported LD_50_ values that ranged from 2.4 to 16.5 times higher for the GSH and Cys conjugates of MCLR and MCYR after intravenous administration in mice, indicating decreased potency for the metabolites [83]. Although microcystin metabolites are less toxic, more research is needed to understand in vivo human liver concentrations of microcystins and their metabolites. 

### 3.4. Excretion

Microcystins are excreted predominately as the parent form into the urine and feces, although multiple studies demonstrated only a small percentage of the dose is excreted from the body [74,83,87]. As discussed above, Robinson et al. reported 83% of the total dose accumulated in the liver and that 24% of the radiolabeled MCLR was excreted in urine (~9%) and feces (~15%) [74]. Most of the urinary excretion (74%) occurred during the first 12 h, with approximately 63% corresponding to the parent toxin and the remaining 30% as metabolites. The rate of excretion through feces was 0.9% and 0.5% per hour for the 6-h and the 12-h time points, respectively, which then slowed to 1% per day through 6 days. Another study reported 1.9% of the dose was excreted in the urine 120 min after intravenous microcystin administration [73]. Several other studies have confirmed that the parent form is the most abundant and the Cys conjugate is the next most abundant [83,87] indicating that the GSH conjugate of microcystins is an intermediate form that is readily converted to the Cys conjugate or back to the parent form. 

Fecal excretion after intravenous or intraperitoneal administration indicates biliary excretion of microcystins. Indeed, 1.7% of radiolabeled MCLR was excreted into the bile during a 60 min liver perfusion [88]. In this study, most of the radiolabeled MCLR in the bile and the perfusate were associated with the parent compound, whereas ~85% of the toxin in the liver corresponded to a more polar form of the toxin. In addition, another study reported an average MCLR biliary clearance of 0.019 µL/min/kg after intravenous administration in healthy rats [70]. Finally, intraperitoneal injection of 75% LD_50_ MCLR resulted in time-dependent induction of apoptosis in the small intestine of mice, indicating MCLR biliary excretion [89]. Considering the low amount of urinary and fecal excretion for microcystins, enhancing metabolism and/or excretion could be a strategy to reduce systemic exposure and toxicities after exposure. 

## 4. Microcystin Molecular Toxicology

**Figure 3:**
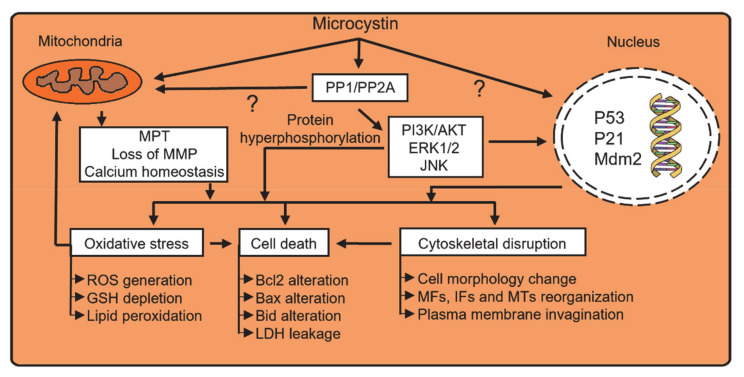
Microcystin molecular toxicity in mammalian cells. Arrows with question marks represent pathways that require further investigation. Abbreviations: PP1: protein phosphatase 1; PP2A: protein phosphatase 2A; PI3/AKT: phosphoinositide 3-kinase/protein kinase B; ERK1/2L extracellular signal-regulated kinases 1/2; JNK: Jun N-terminal kinases; MPT: mitochondrial permeability transition; MMP: mitochondrial membrane potential; P53: tumor promoter p53; P21: cyclin-dependent kinase inhibitor 1; Mdm2: mouse double minute 2 homolog; ROS: reactive oxygen species; GSH: glutathione; Bcl2: B-cell lymphoma 2; Bax: bcl2-associated X protein; Bid: BH3 interacting-domain death agonist; LDH: lactate dehydrogenase; MFs: microfilaments; Ifs: intermediate filaments; MTs: microtubules.

Molecular toxicology of microcystins has been extensively reviewed previously [23,26,90]. The major areas discussed herein, protein phosphatases, oxidative stress, cell death, and cytoskeleton disruption, are interconnected but also play specific roles in microcystin molecular toxicology. Microcystin-mediated genotoxicity is another important molecular effect after microcystin exposure [91], but this will not be reviewed herein. This section will briefly summarize the major molecular toxicological mechanisms for microcystins and identify areas where more research is needed (Figure 3). 

### 4.1. Protein Phosphatases

Microcystins covalently bind to the catalytic subunit of serine/threonine protein phosphatase 1/2A (PP1 and PP2A) enzymes [92,93]. Microcystins are potent inhibitors of PP1 and PP2A, with IC_50_ values ranging from 0.1–1 nM [94,95]. However, different microcystin variants inhibit the protein phosphatases with varying potencies, and are often affected by the methylation/demethylation status of MeAsp and Mdha and also by the amino acid present at position X (Figure 1) [96,97]. Crystal structure analysis of the microcystin-PP1/PP2A complex discovered that microcystins bind to the catalytic site of the enzyme through a two-step process [28,98]. First, the Adda residue of the microcystin interacts with the catalytic subunit of the protein phosphatase enzyme by forming a reversible hydrogen bond. Second, the Mdha residue forms an irreversible covalent bond with a nucleophilic site of the enzyme [23,99,100]. Microcystin metabolism through GSH conjugation prevents covalent binding to protein phosphatases, thus partially explaining the reduced toxicity of microcystin metabolites [100].

Serine/threonine phosphatases are critical regulators of many biological processes such as early embryonic development, cell proliferation, apoptosis, and cancer, and they function to counteract the activity of thousands of protein kinases [101,102]. Downstream targets of PP1/PP2A include the phosphoinositide 3-kinase (PI3K)/AKT and mitogen-activated protein kinase pathways (ERK1/2, JNK, and p38). Studies in HL7702 hepatocytes and mice indicate MCLR dysregulated PI3K/AKT and MAPK pathways and increased the phosphorylation of AKT, ERK1/2, JNK, and p38 [103,104,105,106]. Disruption of protein phosphatase activity by microcystins is linked to many of the other molecular events associated with microcystin toxicity, such as oxidative stress, cell death, and cytoskeleton disruption (see Section 4.2, Section 4.3, Section 4.4). 

Multiple different molecular weights of PP2A were observed after in vivo and in vitro MCLR treatment via Western blot analysis, although not all bands corresponded to MCLR bound PP2A [87,107,108]. Multiple molecular weights were also detected for protein bound MCLR after MCLR treatment [87,107,108]. Not much is known about these different molecular weights of PP2A, and protein bound MCLR, and more research is needed to determine whether they impact PP2A function and microcystin toxicity. 

### 4.2. Oxidative Stress

Oxidative stress, or the imbalance in production and removal of reactive oxygen species (ROS), is a hallmark of microcystin toxicities. Microcystin-elicited oxidative stress is often characterized by changes in lactate dehydrogenase (LDH) leakage, malondialdehyde production (lipid peroxidation), ROS generation, and GSH depletion [109,110]. Early studies in primary rat hepatocytes treated with lyophilized freshwater cyanobacteria reported a time-dependent increase in LDH leakage and ROS production [111]. MCLR treatment in rat hepatocytes caused mitochondrial permeability transition (MPT) along with the loss of the mitochondrial membrane potential (MMP) [112]. This disruption of the electron transport chain increased ROS production and contributed to apoptosis and cytotoxicity [110,112]. A study using HepG2 cells postulated that upregulation of CYP2E1 is a possible source of increased ROS generation because treatment with CYP2E1 inhibitors decreased ROS generation and MCLR cytotoxicity [113]. Another study in HepG2 cells showed that the free radicals produced during oxidative stress caused DNA strand breaks and purine oxidation [114]. Several other in vitro studies in multiple cell lines confirmed ROS generation after microcystin exposure [115,116,117,118]. A bi-phasic response to MCLR treatment was observed in Caco-2 cells, where catalase, GSH peroxidase, and superoxide dismutase activities increased at low concentrations but decreased at high concentrations [118]. These data indicate multiple different components of oxidative stress (ROS production, mitochondrial damage, DNA damage, and enzyme modulation) are involved in microcystin-mediated oxidative stress.

Multiple in vivo studies have also documented the role of oxidative stress in microcystin toxicity. Weng et al. observed that intraperitoneal injection of 60 µg/kg MCLR in mice increased ROS and caused MMP loss in the liver [119]. Increased liver malondialdehyde concentration was observed in rats after 28 days of 32 µg/kg/day MCLR exposure through osmotic pumps [120]. A potential link between JNK activation and ROS generation after MCLR toxicity was suggested in mice, where MCLR-mediated cell death and mitochondrial dysfunction were blocked with *N*-acetylcysteine or JNK inhibitor treatment [121]. Acute MCLR exposure at high doses (100–150 µg/kg intraperitoneal) in rats decreased activities of GSH reductase (60% in liver; 22% in kidney), GSH peroxidase (50% in liver; 31% in kidney), superoxide dismutase (45% in liver; 42% in kidney), and catalase (31% in liver; 28% in kidney), which is consistent with observations of high dose MCLR in Caco-2 cells causing decreased activities of these enzymes [118]. Decreased antioxidant enzyme activity was accompanied by increased lipid peroxidation in the liver (196%) and kidney (58%) [122]. Not surprisingly, pretreatment with oral antioxidants like vitamin C or vitamin E reduced ROS generation and liver injury [119]. Several other studies also reported protection from microcystin mortality when animals were treated with antioxidants such as vitamin E, silymarin, GSH, or monoethyl ester GSH [110,123,124]. GSH depletion in rats increased susceptibility to elevated hepatic malondialdehyde levels but had no appreciable effect on hepatic GSH peroxidase or GSH reductase activities [85]. Although oxidative stress is an important pathological response to microcystin toxicity, conflicting data exist regarding a cause versus effect of its role in microcystin toxicity. 

### 4.3. Cell Death

Depending on the dose, microcystins induce autophagy, apoptosis, and/or necrosis [26,109,112]. In HepG2 cells, non-cytotoxic concentrations of MCLR increased expression of the DNA repair genes *p53*, *p21*, and *Mdm2*, and the pro-apoptotic gene *Bax*, although there was no change in expression of the anti-apoptotic gene *Bcl2* [125]. In Vero-E6 and HepG2 cells, autophagy was observed at doses below the cytotoxic threshold (50 µM for Vero-EG and 25 µM for HepG2), whereas equal proportions of apoptosis and necrosis were observed above the cytotoxic threshold [126]. Autophagy has not been reported in vivo, but apoptosis and necrosis are common after microcystin exposure. For example, Yoshida et al. showed intraperitoneal MCLR caused centrilobular hemorrhage, necrosis, and apoptosis [127]. 

The mechanisms for microcystin-induced cell death involve protein phosphatase inhibition, ROS generation, and disruption of the cytoskeleton and calcium homeostasis [119,120,121,128]. For example, microcystin treatment activated *Bid* and induced apoptosis in hepatocytes through JNK activation [121]. Chen et al. further demonstrated through transcriptomic, proteomic, and simulation strategies that low doses of MCLR induced apoptosis primarily through the Bid-Bax-Bcl2 pathway, whereas high doses of MCLR induced apoptosis through the ROS pathway [129]. Zhao et al. reported that the induction of apoptosis by MCLR involved the disruption of calcium homeostasis through mitochondrial disruption, activation of unfolded protein response, and endoplasmic reticulum stress [130]. In addition, inhibition of calcineurin, a calcium-activated phosphatase, decreased MCLR-mediated apoptosis and necrosis, supporting the role of calcium homeostasis in microcystin-mediated cell death [131].

### 4.4. Cytoskeletal Disruption

Microcystins disrupt cytoskeletal dynamics and change cell morphology. Multiple studies have demonstrated that microcystin toxicity reorganized microfilaments, intermediate filaments, and microtubules leading to deterioration in cell morphology and intercellular interactions [132,133,134]. MCLR toxicity in rat liver caused rearrangement and aggregation of filamentous actin, which disrupted the cell structure and caused hepatocyte dissociation, plasma membrane invagination, and loss of microvilli [135]. Chronic eight-month exposure to MCLR and MCYR in rats caused cytoplasmic aggregation of actin filaments in renal proximal tubule cells [136]. In vitro experiments in rat hepatocytes demonstrated membrane blebbing and condensed microfilaments within 5 min of MCLR treatment without altered intracellular GSH or calcium homeostasis [134]. Cytoskeletal rearrangement was also observed in several other cell types including rat kidney and skin fibroblasts and human epidermal skin [46,137].

Microcystin-elicited cytoskeletal disruption occurs through several mechanisms: (i) altered expression of cytoskeletal genes, (ii) hyperphosphorylation of cytoskeletal proteins, and (iii) ROS generation [24,138,139]. Evidence from an in vivo rat study indicates MCLR treatment decreased transcriptional levels of β-actin (microfilament) and β-tubulin (microtubule) although no significant alteration was observed for vimentin (intermediate filament) levels in the testes [140]. Repeated MCLR exposure in mice increased expression of multiple cytoskeleton organization and actin-binding genes in the liver [139]. In vitro MCLR toxicity caused ROS-mediated activation of the p38 pathway and hyperphosphorylation of the tau protein, the latter being an important regulator of microtubule dynamics [117]. Another study suggested that in vitro microcystin-elicited cytoskeleton disruption was caused by altered protein phosphorylation [137]. ROS are known to directly affect cytoskeleton dynamics and block microcystin-elicited ROS generation through a superoxide dismutase mimic protected against cytoskeleton disruption [133]. 

## 5. Microcystin Pathophysiology

**Figure 4:**
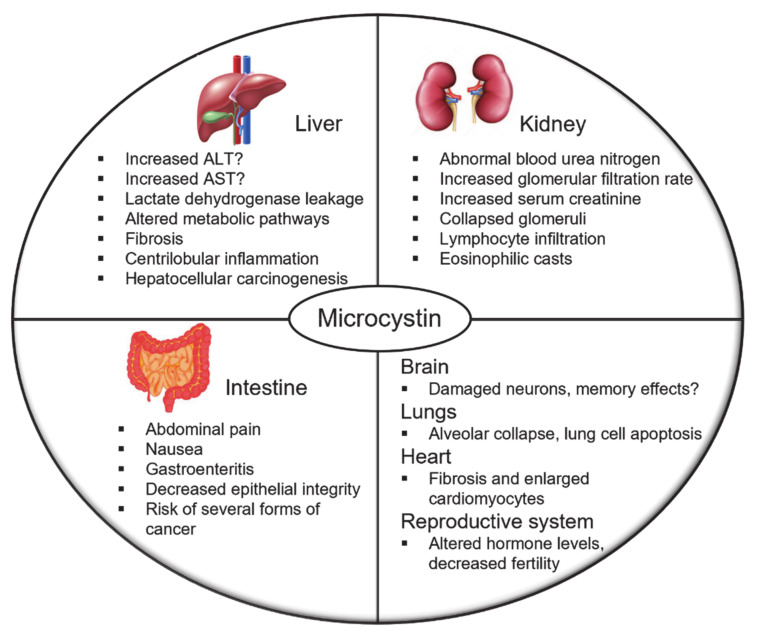
Microcystin pathophysiology in liver, kidney, intestine, brain, lung, heart, and reproductive system. Abbreviations: ALT: alanine aminotransferase; AST: aspartate transaminase. Question marks represent mechanisms that require further investigation.

Abundant data are available for microcystin pathophysiology for multiple organs. Most of the mechanistic data come from preclinical rodent species, but there is an increasing number of epidemiological studies and case reports linking human microcystin exposure to specific pathologies. The following section provides examples of major mechanistic features observed in preclinical models and epidemiological connections to human disease (Figure 4) but is not intended to cover all publications in these areas. 

### 5.1. Liver

As discussed above, the liver is the primary target organ for microcystin toxicity [59,141]. Multiple epidemiological studies found a positive relationship between microcystin concentrations and serum liver damage biomarkers, verifying an increase in liver damage in both adults as well as children, along with increased risks of hepatocellular carcinoma [5,53,57,142]. In preclinical models, acute exposure to microcystins produced centrilobular inflammation, disruption of hepatic plates, apoptosis, and necrosis, which is consistent with observations from the aforementioned dialysis patients from Brazil exposed to microcystin through contaminated dialysate [70,127,143]. Centrilobular fibrosis as a result of microcystin toxicity has been observed in preclinical models as well as in human populations [52,144]. Microcystin-elicited liver damage in preclinical models caused dysfunction in glucose, triglyceride, lipid, and cholesterol metabolic pathways [108,145,146]. Unfortunately, serum biomarkers of liver damage can be unreliable, and preclinical microcystin data suggest inconsistencies in alanine aminotransferase (ALT) and aspartate transaminase (AST) levels after microcystin toxicity [147]. It is unclear whether these inconsistencies are species-dependent and what implications they have for the interpretation of epidemiological data reliant on these biomarkers for assessment of microcystin health effects. 

MCLR is categorized as a possible human carcinogen by the International Agency for Research on Cancer. Preclinical data have demonstrated MCLR acts as a potent tumor promoter after diethylnitrosamine treatment, and sub-lethal MCLR exposure caused neoplastic nodules without the use of an initiator [148,149]. The most robust connection between microcystin exposure and hepatocellular carcinoma was demonstrated in a case-control study in southwest China where serum MCLR concentration quartiles were associated with increased adjusted odds ratios (Q2: 1.3, Q3: 2.6, Q4: 4.0) [54]. Another study found that serum MCLR levels were correlated with increased risk of tumor relapse and overall death after hepatocellular carcinoma-related hepatectomy [150].

### 5.2. Kidney

A comprehensive review of microcystin nephrotoxicity was recently published [151]. Chronic exposure to MCLR in drinking water for six months in mice decreased blood urea nitrogen and caused enlarged renal corpuscles, widened kidney tubules, and increased lymphocyte infiltration [152]. Another study in rats demonstrated that chronic intraperitoneal exposure to MCLR or MCYR for eight months caused glomeruli to collapse and fill with eosinophilic material, and tubules in the outer and inner medulla to dilate and fill with proteinaceous casts [153]. A sub-chronic exposure to MCLR in rats induced similar damage to glomeruli and produced proteinaceous casts, in addition to increased urine volume, proteinuria, and urinary KIM-1 [87]. Similarly, MCLR treatment in perfused rat kidneys increased urine flow, altered glomerular filtration rate, and increased protein material in the urine [154]. Epidemiological data for microcystin effects on renal function and contribution to kidney diseases are limited. In one study, microcystin EDI was determined based on MCLR quantification in water and aquatic products and a study questionnaire. The authors reported that the highest odds ratios for increased blood urea nitrogen and serum creatinine and decreased estimated glomerular filtration rate was observed in the highest MCLR EDI quartile group [155].

### 5.3. Intestine

A recent article has consolidated a detailed review on microcystin intestinal toxicity [128]. Gastroenteritis is the most common acute toxicity after oral microcystin exposure through contaminated food and water or recreational activities [14,32,156,157]. Numerous groups have studied the effect of microcystin exposure and gastrointestinal toxicity [158]. In vitro data in IEC-6 cells indicate MCLR decreased transepithelial electrical resistance mediated by reduced expression of the tight junction proteins occludin and zonula occluden-1, potentially affecting epithelial integrity [159]. In mice, MCLR caused extensive erosion of the villi in the small intestine, which the authors suggested allowed more toxin to pass the barrier of the villi [71]. Ito et al. reported an age-dependent susceptibility to intestinal damage, where older mice were more susceptible to MCLR-elicited toxicity [160]. 

Another important aspect of intestinal pathophysiology is the gut microbiota. Sub-chronic exposure to MCLR increased microbial species richness in the caecum and colon and diversity in the caecum [161]. Most of the microbes that decreased were *Lachnospiraceae* and those that increased were *Porphyromonadaceae* [161]. There have been no reports of microbiome effects in humans. 

An extensive review on incidences of acute and chronic cyanobacterial exposure in people was published recently [158]. Acute exposure to microcystin contaminated water is associated with gastroenteritis, vomiting, nausea, and diarrhea [162]. Chronic exposure has been correlated to an increased incidence of colorectal cancer [163]. 

### 5.4. Other Organ Targets

Microcystin toxicity has also been reported in the nervous system, lungs, heart, and reproductive system. Fischer et al. reported that human OATP1A2, which is expressed at the blood–brain barrier, transported MCLR [59]. Rodent studies demonstrated that microcystins damaged neurons in the hippocampus and also affected its long-term potential, which is important for learning and memory [131,164,165]. Another in vivo study has also demonstrated that MCLR exposure can cause blood–brain barrier dysfunction by causing neuroinflammatory responses and microglial and astrocyte impairment [166]. MCLR can also induce tau hyperphosphorylation, spatial memory impairment, neuronal degenerative changes that can contribute to Alzheimer’s disease [167]. In mouse lungs, chronic MCLR exposure for six months induced alveolar collapse, apoptosis, and disrupted cell junction integrity [168]. Li et al. demonstrated that a 12-month exposure to MCLR increased lung inflammatory cytokines and caused alveolar septa thickening in mice [169]. Cardiac toxicity may also occur after microcystin exposures. Chronic exposure to MCYR for eight months in rats decreased the volume density of cardiac muscle tissues because of increased fibrosis along with increased lymphocyte infiltration and enlarged cardiomyocytes [170]. Another study in rats that were administered a lethal dose of a microcystin mixture from cyanobacterial extract reported loss of adherence between cardiomyocytes and swollen and ruptured mitochondria, which the author suggested may contribute to death in severe microcystin intoxication cases [171]. Finally, a number of in vivo studies have associated microcystin toxicity with decreased testosterone levels, sperm quality, ovarian damage, and decreased female fertility [140,172]. 

## 6. Microcystins in the Multifactorial Etiology of Chronic Diseases

**Figure 5:**
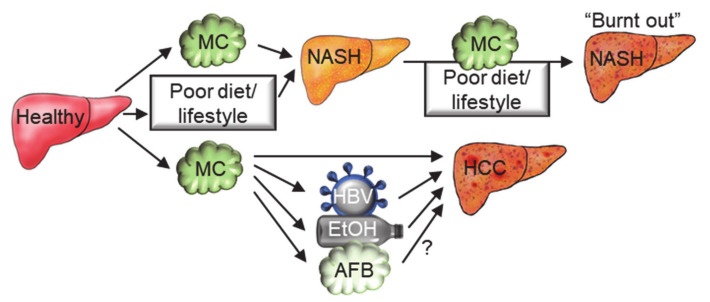
Role of microcystins in multifactorial etiology for NASH and HCC. Arrows with question marks represent pathways that require further investigation. Abbreviations: MC: microcystin; NASH: nonalcoholic steatohepatitis; HBV: hepatitis B virus; EtOH: ethanol; AF: aflatoxin.

Microcystins are only one of many toxic insults people are exposed to that can contribute to disease pathogenesis and/or progression. Recent evidence suggests that exposure to microcystins in conjunction with other hepatotoxic factors can have combined effects on disease. Microcystin involvement in nonalcoholic fatty liver disease (NAFLD), hepatocellular carcinoma, and chronic kidney disease are discussed in this section (Figure 5). 

NAFLD has a global prevalence of approximately 25% and is one of the most common chronic liver diseases worldwide [173,174]. Nonalcoholic steatohepatitis (NASH) is an advanced stage of NAFLD that can lead to cirrhosis and hepatocellular carcinoma [174]. Key factors that contribute to the pathogenesis and progression of NAFLD include chronic hepatic stress from a poor diet/lifestyle and exposure to exogenous hepatotoxins [108,175,176]. It was previously demonstrated that chronic low dose MCLR exposure promoted a pathology similar to NASH in mice [176,177]. In addition, two other studies demonstrated that diet-induced or genetic models of NAFLD altered MCLR toxicokinetics with higher and longer systemic exposure to MCLR in NAFLD compared to healthy controls [70,178]. An ecological study using satellite imaging techniques further showed that there is a direct correlation between incidences of microcystin producing cyanobacteria blooms and NAFLD [179]. Regarding disease severity, four weeks of intraperitoneal MCLR exposure in preexisting NASH created a more severe liver phenotype that resembled burnt-out NASH, which is an important risk factor for NAFLD progression [108]. Another group demonstrated that four weeks of oral exposure to MCLR in mice exacerbated overall liver injury and caused genetic and phosphoproteomic dysregulation of key signaling pathways in NAFLD [178]. These data for the role of microcystins in NAFLD pathogenesis and progression have all been in rodent species, and more research is needed to assess the potential risk microcystins may pose to people with preexisting NAFLD. 

Co-exposure to microcystins and other factors such as aflatoxin, hepatitis B virus infection, and alcohol have been investigated in hepatocellular carcinoma and kidney disease. A cross-sectional study that estimated MCLR and aflatoxin exposures through the diet reported an increased odds ratio for abnormal AST and ALT in subjects with hepatitis B virus infection, aflatoxin exposure, and MCLR exposure [180]. In contrast, a case-control study reported positive interactions for hepatocellular carcinoma between MCLR serum levels and hepatitis B virus infection or alcohol consumption (synergism index of 3.0 and 4.0, respectively) but a negative interaction between serum levels of MCLR and aflatoxin-albumin adduct (synergism index of 0.4) [54]. Although an in vitro assessment in human liver cell lines suggested that MCLR may increase aflatoxin DNA damage, a follow-up study in rats confirmed the finding in humans by demonstrating that MCLR co-exposure reduced aflatoxin-mediated carcinogenesis [181,182]. Finally, although MCLR was reported to decrease renal function in a population in rural southwest China, no combined effect of MCLR and aflatoxin was observed [155]. Overall, these data indicate co-exposure of microcystins with a NAFLD diet/lifestyle, hepatitis B viral infection, or alcohol consumption could increase the risk of exacerbating NAFLD and/or inducing hepatocellular carcinoma. 

## 7. Conclusions

This review has compiled data for microcystin exposure, toxicokinetics, and its associated toxicities. It is known that human microcystin exposure occurs throughout the world and results in detectable systemic microcystin concentrations and various organ toxicities. A major gap that still exists is the lack of a clear understanding of human toxicokinetics of microcystins, including multiple aspects of absorption, distribution, metabolism, and excretion. More research is needed to understand the fraction of microcystin that is absorbed and tissue-specific distribution in humans. Another important area of future research is to develop a better understanding of microcystin co-exposure with other toxic insults and how these factors contribute to the development of chronic diseases. Exposure to multiple pathogenic factors such as poor diet/lifestyle and exogenous toxins affect complex molecular and cellular mechanisms that can compound the risk of some diseases. Repeated exposure to such factors in populations with underlying disease conditions, such as NAFLD, can also exacerbate disease, leading to HCC. Addressing these gaps can inform microcystin exposure limits and risk assessment and improve public health. 

## Figures and Tables

**Table 1:** Principal groups of cyanotoxins, their producing genera, and the toxicities associated with them (Information compiled from [3,14,16,21]).

Toxin	Producing Genera	Primary Toxicity	Mode of Action	Toxic Effects
Microcystin	*Microcystis, Anabaena, Nostoc, Planktothrix, Hapalosiphon, Phormidium*	Hepatotoxicity	Inhibition of protein phosphatases	Liver and kidney damage, gastroenteritis, tumor promotion, reduced DNA repair, and reproductive toxicity
Nodularins	*Nodularia*	Hepatotoxicity	Inhibition of protein phosphatases	Liver and kidney damage, gastroenteritis, tumor promotion, reduced DNA repair, and reproductive toxicity, carcinogenic
Cylindrospermopsins	*Cylindrospermopsis, Anabaena, Raphidiopsis, Aphanizomenon, Chrysosporum, Umezakia*	Hepatotoxicity	Inhibition of protein phosphatases	Liver, kidney, spleen, lungs and intestinal damage, genotoxicity
Anatoxin-a	*Anabaena,* *Aphanizomenon, Cuspidothrix, Dolichospermum, Oscillatoria, Phormidium*	Neurotoxicity	Nicotinic acetylcholine receptor agonists	Muscular paralysis, respiratory failure
Anatoxin-a(s)	*Dolichospermum, Anabaena*	Neurotoxicity	Inhibition of acetylcholinesterase	Muscular weakness, dyspnea, convulsions
Saxitoxins	*Aphanizomenon, Cuspidothrix, Cylindrospermopsis, Dolichospermum*	Neurotoxicity	Blocking of sodium channels	Convulsions, paralysis, respiratory failure
BMAA (β-Methylamino-L-alanine)	*Microcystis, Nostoc, Anabaena, Aphanizomenon, Nodularia*	Neurotoxicity	Excessive stimulation of glutamate receptors in neurons	Neurodegenerative syndrome
Aplysiatoxin	*Lyngbya, Schizothrix, Oscillatoria*	Dermatoxicity	Activation of protein kinase C	Tumor promotion, skin irritation, asthma
Lyngbyatoxins	*Lyngbya, Schizothrix, Oscillatoria*	Dermatoxicity	Activation of protein kinase C	Tumor promotion, skin and eye irritation, respiratory problems
Lipopolysaccharide	All cyanobacteria	Dermatoxicity	Activation of toll-like receptors	Skin and eye irritation, fever, gastrointestinal upset

## Data Availability

No new data were created or analyzed in this study. Data sharing is not applicable to this article.
